# Assessment of organ doses, peak skin doses and effective doses in patients undergoing anterior cervical discectomy and fusion utilising VirtualDose-IR software

**DOI:** 10.1093/rpd/ncad286

**Published:** 2023-11-28

**Authors:** Vasileios Metaxas, Christos Dimitroukas, Fotios Efthymiou, Harry Delis, George Gatzounis, Fotios Tzortzidis, Petros Zampakis, Andreas Theofanopoulos, Constantine Constantoyannis, George Panayiotakis

**Affiliations:** Department of Medical Physics, School of Medicine, University of Patras, 26504 Patras, Greece; Department of Medical Physics, School of Medicine, University of Patras, 26504 Patras, Greece; Department of Medical Physics, University Hospital of Patras, 26504 Patras, Greece; Department of Medical Physics, School of Medicine, University of Patras, 26504 Patras, Greece; Department of Medical Physics, School of Medicine, University of Patras, 26504 Patras, Greece; Department of Neurosurgery, University Hospital of Patras, 26504 Patras, Greece; Department of Neurosurgery, School of Medicine, University of Patras, 26504 Patras, Greece; Department of Neurosurgery, University Hospital of Patras, 26504 Patras, Greece; Department of Radiology, University Hospital of Patras, 26504 Patras, Greece; Department of Radiology, School of Medicine, University of Patras, 26504 Patras, Greece; Department of Neurosurgery, University Hospital of Patras, 26504 Patras, Greece; Department of Neurosurgery, University Hospital of Patras, 26504 Patras, Greece; Department of Neurosurgery, School of Medicine, University of Patras, 26504 Patras, Greece; Department of Medical Physics, School of Medicine, University of Patras, 26504 Patras, Greece; Department of Medical Physics, University Hospital of Patras, 26504 Patras, Greece

## Abstract

In this study, the effect of patient- and procedure-related parameters on organ doses (ODs), peak skin dose (PSD) and effective dose (*E*) during anterior cervical discectomy and fusion (ACDF) was evaluated. Patient- and procedure-related parameters, as well as fluoroscopy time, kerma-area product (KAP), cumulative air-kerma (*K*_air_) and incident *K*_air_, were analysed for 50 ACDF procedures performed with a mobile C-arm. These parameters were inserted in VirtualDose-IR software implementing sex-specific and body mass index (BMI)-adjustable anthropomorphic phantoms to calculate OD, PSD and *E*. The BMI, gender and type of implants did not significantly affect KAP, incident *K*_air_, PSD and *E*. However, the type of fusion significantly affected the *E*. The single fusions in C5/C6 resulted in significantly higher KAP, incident *K*_air_ and *E* than C4/C5 levels, while those performed in C6/C7 resulted in significantly higher *E* and PSD than C4/C5 levels. The thyroid, oesophagus and salivary glands received the largest doses in all groups studied. The BMI did not significantly affect ODs. The salivary glands absorbed significantly higher doses in males than females, while the extrathoracic region’s dose significantly increased for multi- than single-level fusions. The fusions in C6/C7 resulted in significantly higher oesophagus and thyroid doses than C3/C4 and C4/C5 levels, as well as fusions performed in C5/C6 compared with C4/C5 levels. The data presented here could be used by the neurosurgeons as a comparator for future studies in optimising radiation protection during ACDF procedures in the operating theatre by keeping the ODs, PSD and *E* as low as reasonably practicable.

## Introduction

Anterior cervical discectomy and fusion (ACDF) is a type of spine surgery where a herniated disc is removed to treat spinal cord or nerve root pressure and relieve the resulting pain, weakness, numbness and tingling. Additionally, ACDF is performed to treat cervical degenerative disc disease, remove osteophytes associated with arthritis and relieve cervical spinal stenosis symptoms^(1)^. Neurosurgeons use fluoroscopy to guide and confirm the accurate and safe placement of the instrumentation. However, this results in exposure to ionising radiation of patients and medical staff inside the operating theatre^(2)^. Thus, monitoring radiation dose and optimising these procedures is crucial to minimise the potential for radiation-induced effects^(3–5)^. For radiation dose management during interventional procedures, it is important to provide information regarding the kerma-area product (KAP), fluoroscopy time (FT), cumulative air-kerma (*K*_air_) (at the interventional reference point (IRP)), entrance surface dose (ESD), organ doses (ODs) and effective dose (*E*)^(2, 6)^.

Several studies deal with radiation dose evaluation during cervical spine interventional procedures performed with a C-arm^(7–15)^. Most commonly, the FT, KAP and cumulative *K*_air_ were obtained from the dosimetric report of the C-arm^(7, 8, 11–13, 15)^. The ESD values were calculated based on exposure parameters utilising a mathematical equation^(15)^, while the *E* values were estimated from the recorded KAP values utilising suitable conversion coefficients^(7, 11, 13)^. Another method to calculate *E* and OD values is utilising Monte Carlo (MC) simulations in stylised or anthropomorphic phantoms^(7, 12)^, considering clinical exposure parameters. Experimental measurements of patients’ ESD were also performed, utilising a polymethyl-methacrylate (PMMA) phantom^(14)^ or a cervical cadaveric specimen simulating the patient during clinical exposure^(9, 10)^. The ESD could also be calculated by utilising KAP and the corresponding field size at a specific focus-to-skin distance (FSD)^(12)^. However, for cervical spine interventions, which include irradiation of a small part of the human body, the *E* is considered a less appropriate indicator of radiation-induced stochastic risk of an individual than a suitable set of ODs. The thyroid is the most radiosensitive organ in this region, and the thyroid dose should be considered, especially when young patients are involved, given that the excess risk of thyroid cancer persists for at least four decades after exposure. Nevertheless, only a few data are available regarding ODs during cervical spine interventions^(12, 13)^.

It has been found that the PMMA phantom thickness across the X-ray beam and the operators’ selection of the C-arm’s technical settings (continuous and/or high-dose fluoroscopy and use of electronic magnification) are associated with a higher radiation dose^(14)^. Generally, patients with greater body mass index (BMI) receive more radiation dose in the lumbar spine^(12, 16, 17)^ and cervical spine surgery^(12, 13)^ due to the greater radiation exposure necessary to produce an acceptable image. In a previous study, it has also been demonstrated that single-level procedures in C1-C2-C3-C4-C5 require significantly lower KAP compared with those in the C5-C6-C7 levels^(13)^. Additionally, relatively lower KAP values were reported for females compared with males, for single- compared with multiple-level fusions, and in cases where a cage was used as a stand-alone device^(13)^. Although patients’ BMI and gender, cervical levels, type of fusion (single- or multiple-level) and type of implants (cages or cages plus plates and screws) affected dose indices (KAP and *K*_air_), there is no data regarding their effect on OD and *E* during ACDF procedures.

This study aimed to evaluate the influence of patient- (BMI and gender) and procedure-related data (type of fusion, cervical levels and type of implants) on OD, peak skin dose (PSD) and *E* during ACDF procedures utilising VirtualDose-IR, an MC-based software implementing sex-specific and BMI-adjustable anthropomorphic phantoms.

## Materials and methods

### Patients and ACDF procedure

Fifty patients suffering from cervical disc herniation and osteophytes, degenerative disc disease, myelopathy and spine fractures underwent ACDF at the University Hospital of Patras’ Neurosurgery Department between May 2020 and March 2021. Ethics approval was obtained by the Hospital’s ethics committee. All ACDF procedures were performed by the same surgical team (a senior neurosurgeon assisted by a trainee) without providing any practical guidance to the neurosurgeon regarding the manipulation of the C-arm system. If a procedure differed from the standard routine practice, it was excluded from the study. The surgical technique has been described in detail in previous studies^(12, 13)^. An anterior approach was implemented. Cages for interbody fusion were utilised either alone or in combination with plates and screws. A right lateral X-ray projection was acquired with the patient in a supine position and the X-ray tube adjacent to the neurosurgeon on the right side of the operating table.

For each procedure, patient-related data (age, gender, weight, height, BMI), procedure-related data (tube voltage, cervical levels, type of fusion (single- or multi-level fusion)), type of implants (cages or cages plus plates and screws), as well as the FT, KAP and cumulative *K*_air_ from the dosimetric report of the C-arm were recorded.

Patients were categorised into different groups based on their BMI values: normal (18.5–24.9 kg m^−2^), overweight (25–29.9 kgm^−2^) and obese (>30 kgm^−2^)^(18)^; gender: males and females; type of fusion: single- or multi-level fusion; cervical levels (for single-level fusions): C3/C4, C4/C5, C5/C6, C6/C7; and type of implants used (cages or cages plus plates and screws), to assess their influence on OD, PSD and *E* values.

### C-arm system and exposure parameters

All ACDF procedures were performed using a mobile C-arm (Philips BV Endura, Philips Healthcare, Eindhoven, The Netherlands) utilising the exposure and operative parameters in Table 1. A conventional 23-cm image intensifier was implemented in the C-arm. The focus-to-detector distance (FDD) is fixed at 100 cm. Three fields of view (FOVs) were available with 23-, 17- and 14-cm diameter sizes. The ‘Head/Spine’ acquisition protocol was used. Automatic brightness control (ABC) was used to adjust tube voltage and tube current to provide an image of clinically acceptable quality regarding the patient’s specific anatomical characteristics. The C-arm provided a dose report including FT, KAP and cumulative *K*_air_ values. The inverse square law was applied to correct the differences between the IRP and the actual FSD. The incident *K*_air_ was calculated by multiplying the cumulative *K*_air_ values with the correction factor. The FSDs were measured during half of the procedures and were found between 54 and 58 cm with a mean value of 56 cm. The patient’s head was fixed in all cases with a Mayfield mechanism attached to the table, while the table height did not affect the patient and C-arm system set-up. Due to the small range, we use a fixed FSD of 56 cm during all simulations.

**Table 1:** Exposure and operative parameters obtained from the ACDF procedures used as input in the VirtualDose-IR software for the dose calculations

Exposure and operative parameters	
Patient phantom	Male: normal, overweight, obese
	Female: normal, overweight, obese
Projection region on the phantom	As indicated in Figure 1
X-ray projection	Right lateral
FOV (cm)^a^	20.38
Tube voltage (kVp)^b^	70
Additional copper filtration (mm)	0.1
FDD (cm)	100
FSD (cm)	56
Dose calculation type	KAP
Organ weighting scheme	ICRP 103

ICRP: International Commission on Radiological Protection.

^a^Square field at detector plane. This field is equal to the circular field with 23-cm diameter used in clinical practice.

^b^The clinical tube voltages ranged between 59 and 74 kVp with a mean value of 65 kVp. The 70 kVp is the lowest tube voltage value available in VirtualDose-IR software for cervical spine irradiation. This value is as close as possible to those used in clinical practice regarding the options provided by the software.

The C-arm was under a periodic quality control program by the Medical Physics Department of the Hospital to ensure the reliability and reproducibility of its dosimetric and imaging performance. The software KAP meter was calibrated in situ according to the method proposed by IAEA^(19)^. The measurements were performed with a cylindrical ionisation chamber (model 10 × 6-6) and a calibrated RadCal electrometer (Radcal Accu Pro 9096, Monrovia, CA, USA) considering the clinical exposure conditions. A correction factor of 1.28 was applied to the KAP recordings to avoid KAP underestimation and to achieve adequate OD and *E* estimations accuracy. The examinations were mainly carried out using continuous low-dose fluoroscopy, without a magnified FOV or any collimation of the X-ray field with the iris or shutters; however, in some cases, optional settings regarding fluoroscopy (pulsed or high-dose fluoroscopy) and magnified FOVs were used to adjust radiation exposure.

### OD, PSD and *E* calculations

**Figure 1:**
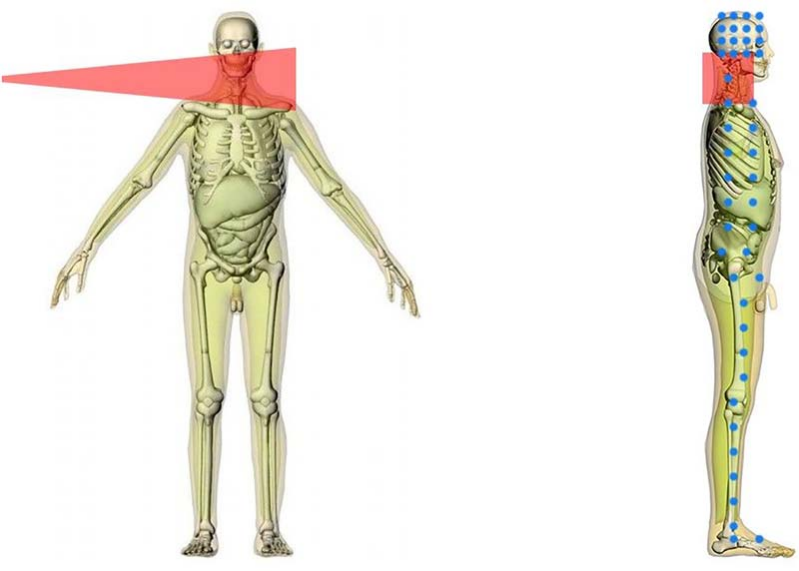
A representative image of the left lateral projection used to irradiate the overweight male phantom during simulation in VirtualDose-IR software of an ACDF procedure. The square X-ray field is defined at the image intensifier surface, and the dot located in the patient’s cervical spine is selected as the entrance point of the X-ray beam

For the calculation of ODs, PSDs and Es, MC simulations were carried out utilising the commercially available web-based software (VirtualDose-IR, Virtual Phantoms Inc., USA)^(20)^ incorporating male and female adult mathematical voxel-based phantoms according to the models provided by ICRP^(21)^. For each patient and each ACDF procedure, MC simulations were carried out by adjusting the phantom according to the patient’s BMI and considering the exposure and operative parameters (X-ray projection, tube voltage, filtration, FDD, FSD and dosimetric quantity) included in Table 1. The clinical tube voltage values ranged between 59 and 74 kVp with a mean value of 65 kVp. The 70 kVp is the lowest tube voltage value available in VirtualDose-IR for cervical spine irradiation. Additionally, the software does not allow users to insert non-round numeric values for tube voltage. Therefore, the simulations were carried out with a tube voltage of 70 kVp to be representative of the tube voltage values applied in clinical practice. The X-ray field was positioned in a lateral projection according to the cervical spine level by visually selecting the corresponding dot in the phantom images (Figure 1), which identifies the centre of the anatomical region. This way, the clinically irradiated area is approximately the same as that used during the simulations. However, the X-ray field position may change during clinical practice, while in the simulations, it is kept fixed. Additionally, the circular X-ray field of 23 cm diameter, which was mainly used in clinical practice, was approximated with a square field of 20.38 cm × 20.38 cm at the detector plane during the simulations (Table 1). The *E* is estimated based on ICRP Publication 103^(22)^ organ and tissue weighting factors.

### Statistical analysis

Descriptive statistics (mean, median, range, interquartile range) were provided for patients’ data, tube voltage, FT, KAP, *K*_air_, *E*, PSD and OD values per BMI group, gender, type of fusion, cervical levels and type of implants used for the ACDF procedures. The normality of the data was examined by the Smirnov–Kolmogorov goodness-of-fit test. All the dosimetric data follow non-normal distribution (*p* < 0.01). Thus, the Mann–Whitney and the Kruskal–Wallis tests were used to investigate the existence of a significant difference among the various groups studied, as appropriate. Additionally, pairwise comparisons were performed between groups using Conover’s post-hoc test to identify the specific pairs that provided strong evidence of a significant difference. The SPSS v.25 statistical package was used for the statistical analysis (IBM Corp, Armonk, NY). Statistics were considered significant at *p* < 0.05.

## Results and discussion

The primary aim of this study was to investigate the effect of patient- and procedure-related data on ODs, PSD and *E* received by patients undergoing ACDF procedures. Of the 50 patients, 32 were males and 18 were females. The total mean age and BMI were 51 years (range, 20–76) and 28.2 kgm^−2^ (range, 21.3–38.2), respectively. The patients’ data are presented in Table 2.

**Table 2:** Patients’ data categorised per BMI, gender, type of procedure, treated levels and type of surgery used during ACDF procedures

Patients’ data^*^	Number of patients (*N*)	Age (years)	Weight (kg)	Height (m)	BMI (kgm^−2^)
Total sample	50	51 (20–76)	82 (56–135)	1.70 (1.51–1.85)	28.2 (21.3–38.2)
BMI					
Normal	9	47 (31–76)	68 (56–78)	1.71 (1.62–1.80)	23.2 (21.3–24.8)
Overweight	30	53 (25–76)	80 (62–100)	1.70 (1.51–1.83)	27.5 (25.0–29.8)
Obese	11	50 (20–74)	102 (79–135)	1.71 (1.57–1.85)	34.3 (30.5–38.2)
Gender					
Female	18	51 (34–75)	75 (56–104)	1.65 (1.51–1.75)	27.3 (21.3–37.3)
Male	32	51 (20–76)	87 (69–135)	1.73 (1.60–1.85)	28.7 (22.5–38.2)
Type of fusion					
Single-level	35	50 (20–76)	83 (56–135)	1.71 (1.51–1.85)	28.2 (21.3–38.2)
Multi-level	15	54 (31–74)	81 (67–104)	1.69 (1.61–1.78)	28.3 (24.0–35.2)
Treated levels					
C3/C4	6	58 (46–75)	79 (62–100)	1.70 (1.51–1.83)	27.5 (23.2–31.6)
C4/C5	13	49 (20–65)	93 (70–135)	1.73 (1.59–1.85)	30.5 (24.8–38.2)
C5/C6	12	50 (25–76)	77 (65–92)	1.70 (1.57–1.83)	26.9 (22.5–37.3)
C6/C7	4	41 (33–46)	75 (56–85)	1.70 (1.62–1.73)	25.8 (21.3–29.1)
Type of implants					
Cages	32	51 (25–76)	81 (56–135)	1.70 (1.51–1.83)	27.9 (21.3–38.2)
Cages, plates, screws	18	51 (20–74)	84 (65–130)	1.71 (1.61–1.85)	28.9 (22.5–37.9)

BMI: body mass index.

^*^Mean values. Range is indicated in parenthesis.

During ACDFs, fluoroscopy is used to localise and guide the positioning of cages, plates and screws^(12)^. The median clinical tube voltage, FT, KAP, cumulative *K*_air_, incident *K*_air_ and *E* values during ACDF procedures are presented in Table 3 as a function of patients’ BMI, gender, type of fusion, cervical levels (for single-level fusions) and type of implants used.

**Table 3:** Dosimetric data categorised per BMI, gender, type of procedure, treated levels and type of surgery used during ACDF procedures

Dosimetric data^*^	Clinical tube voltage (kVp)	FT (s)	KAP (Gycm^2^)	Cumulative *K*_air_ (mGy)^a^	Incident *K*_air_ (mGy)^b^	*E* (mSv)
Total sample	64 (62–66)	5.0 (3.0–9.0)	0.06 (0.03–0.10)	0.26 (0.14–0.46)	0.41 (0.21–0.70)	0.010 (0–0.020)
BMI						
Normal	63 (62–69)	7.0 (3.0–12.0)	0.05 (0.04–0.26)	0.23 (0.18–1.18)	0.35 (0.28–1.82)	0.020 (0.008–0.022)
Overweight	64 (61–65)	5.0 (3.0–9.0)	0.07 (0.03–0.10)	0.30 (0.13–0.44)	0.45 (0.21–0.68)	0.010 (0–0.010)
Obese	65 (63–66)	3.0 (3.0–5.0)	0.03 (0.02–0.10)	0.15 (0.11–0.47)	0.23 (0.18–0.72)	0.010 (0–0.020)
Gender						
Female	64 (63–65)	6.5 (3.0–9.0)	0.06 (0.02–0.12)	0.28 (0.11–0.52)	0.43 (0.16–0.80)	0.010 (0–0.020)
Male	64 (62–66)	5.0 (3.0–8.0)	0.06 (0.03–0.10)	0.25 (0.14–0.45)	0.38 (0.21–0.69)	0.010 (0.005–0.020)
Type of fusion						
Single-level	64 (62–65)	4.0 (3.0–9.0)	0.06 (0.03–0.10)	0.26 (0.14–0.45)	0.40 (0.22–0.70)	0.010 (0–0.018)
Multi-level	64 (61–67)	6.0 (2.0–10.0)	0.07 (0.02–0.13)	0.32 (0.10–0.59)	0.49 (0.16–0.90)	0.010 (0.010–0.020)
Treated levels						
C3/C4	64 (62–65)	2.5 (2.0–4.0)	0.04 (0.02–0.10)	0.16 (0.07–0.44)	0.24 (0.11–0.67)	0 (0–0.010)
C4/C5	64 (62–65)	5.0 (3.0–7.0)	0.04 (0.03–0.06)	0.18 (0.12–0.28)	0.28 (0.19–0.43)	0 (0–0.010)
C5/C6	64 (62–66)	4.0 (3.0–11.0)	0.09 (0.05–0.14)	0.39 (0.23–0.60)	0.60 (0.35–0.92)	0.010 (0.010–0.030)
C6/C7	65 (64–66)	8.0 (6.0–19.0)	0.07 (0.05–0.60)	0.30 (0.23–2.70)	0.45 (0.35–4.17)	0.020 (0.015–0.060)
Type of implants						
Cages	64 (62–65)	4.0 (3.0–7.0)	0.06 (0.03–0.10)	0.25 (0.14–0.44)	0.38 (0.22–0.68)	0.010 (0–0.020)
Cages, plates, screws	64 (61–67)	7.5 (3.0–12.0)	0.07 (0.03–0.11)	0.32 (0.13–0.51)	0.49 (0.21–0.78)	0.010 (0.010–0.020)

*K*_air_: air-kerma.

^*^Median values. Interquartile range is indicated in parenthesis.

^a^Cumulative *K*_air_ is calculated at the IRP.

^b^Incident *K*_air_ is calculated at the actual FSD.

### Tube voltage

No significant differences were found in clinical tube voltage values regarding all the different groups studied (*p* > 0.05) (Table 3). The above is mainly attributed to almost similar anatomical characteristics for all the patients in the neck region. Thus, using a tube voltage of 70 kVp as input in the simulations (Table 1), the obtained dose values (Tables 4–6) are considered to be on ‘the safe side’.

**Table 4:** ODs categorised per BMI and gender of the patients underwent ACDF procedures

Organ	ODs (mGy)					
	Total sample	BMI			Gender	
		Normal	Overweight	Obese	Male	Female
	Median (IQR)	Median (IQR)	Median (IQR)	Median (IQR)	Median (IQR)	Median (IQR)
Bone surface	0.01 (0–0.01)	0.01 (0.01–0.02)	0.01 (0–0.01)	0.01 (0–0.01)	0.01 (0–0.01)	0.01 (0–0.02)
RBM	0.01 (0–0.01)	0.01 (0–0.01)	0.00 (0–0.01)	0.01 (0–0.01)	0.00 (0–0.01)	0.01 (0–0.01)
Lungs	0 (0–0.01)	0.01 (0–0.01)	0 (0–0.01)	0.01 (0–0.01)	0.00 (0–0.01)	0 (0–0.01)
Oesophagus	0.05 (0.03–0.12)	0.12 (0.04–0.14)	0.04 (0.03–0.09)	0.08 (0.03–0.11)	0.04 (0.02–0.11)	0.06 (0.04–0.12)
Salivary glands	0.03 (0.01–0.06)	0.03 (0.02–0.04)	0.04 (0.01–0.06)	0.03 (0.02–0.07)	0.01 (0.01–0.03)	0.06 (0.04–0.10)
Skin	0.01 (0.01–0.02)	0.01 (0.01–0.02)	0.01 (0.01–0.02)	0.01 (0.01–0.02)	0.01 (0–0.02)	0.01 (0.01–0.02)
Thyroid	0.11 (0.06–0.25)	0.24 (0.08–0.31)	0.10 (0.05–0.17)	0.18 (0.06–0.26)	0.10 (0.04–0.26)	0.12 (0.06–0.24)
Extrathoracic region	0.01 (0–0.02)	0.02 (0.01–0.02)	0.01 (0–0.01)	0.01 (0.00–0.02)	0.01 (0–0.02)	0.01 (0.00–0.02)
Muscle	0.01 (0.01–0.02)	0.02 (0.01–0.02)	0.01 (0.01–0.02)	0.01 (0.01–0.02)	0.01 (0–0.01)	0.01 (0.01–0.02)
Oral mucosa	0.02 (0.01–0.03)	0.03 (0.01–0.03)	0.02 (0.01–0.03)	0.02 (0.01–0.04)	0.01 (0.01–0.03)	0.02 (0.01–0.04)

IQR: interquartile range. RBM: red bone marrow.

**Table 5:** ODs categorised per type of fusion and type of implants used during ACDF procedures

Organ	ODs (mGy)			
	Type of fusion		Type of implants	
	Single-level	Multi-level	Cages	Cages, plates and screws
	Median (IQR)	Median (IQR)	Median (IQR)	Median (IQR)
Bone surface	0.01 (0–0.01)	0.01 (0.01–0.02)	0.01 (0–0.01)	0.01 (0.01–0.02)
RBM	0.01 (0–0.01)	0.01 (0–0.01)	0 (0–0.01)	0.01 (0–0.01)
Lungs	0 (0–0.01)	0 (0–0.01)	0 (0–0.01)	0.01 (0–0.01)
Oesophagus	0.04 (0.03–0.10)	0.08 (0.04–0.12)	0.04 (0.02–0.10)	0.08 (0.04–0.12)
Salivary glands	0.03 (0.01–0.06)	0.05 (0.01–0.12)	0.03 (0.01–0.06)	0.04 (0.02–0.09)
Skin	0.01 (0.00–0.02)	0.01 (0.01–0.03)	0.01 (0–0.02)	0.01 (0.01–0.02)
Thyroid	0.11 (0.05–0.20)	0.21 (0.08–0.26)	0.09 (0.05–0.23)	0.18 (0.09–0.26)
Extrathoracic region	0.01 (0–0.02)	0.02 (0.01–0.02)	0.01 (0–0.02)	0.01 (0.01–0.02)
Muscle	0.01 (0–0.02)	0.01 (0.01–0.03)	0.01 (0–0.02)	0.01 (0.01–0.02)
Oral mucosa	0.02 (0.01–0.03)	0.02 (0.01–0.04)	0.02 (0.01–0.03)	0.02 (0.01–0.04)

RBM: red bone marrow.

**Table 6:** ODs categorised per cervical level treated during ACDF procedures

Organ	ODs (mGy)			
	Treated levels			
	C3/C4	C4/C5	C5/C6	C6/C7
	Median (IQR)	Median (IQR)	Median (IQR)	Median (IQR)
Bone surface	0 (0–0)	0 (0–0.01)	0.01 (0.00–0.02)	0.02 (0.01–0.04)
RBM	0 (0–0)	0 (0–0.01)	0.01 (0.00–0.02)	0.01 (0.01–0.04)
Lungs	0 (0–0)	0 (0–0.01)	0.00 (0–0.01)	0.01 (0.00–0.02)
Oesophagus	0.03 (0.03–0.04)	0.03 (0.02–0.04)	0.06 (0.04–0.16)	0.12 (0.08–0.35)
Salivary glands	0.02 (0.01–0.03)	0.02 (0.01–0.04)	0.04 (0.02–0.06)	0.08 (0.04–0.34)
Skin	0.00 (0–0.01)	0.01 (0–0.01)	0.01 (0.01–0.02)	0.02 (0.02–0.08)
Thyroid	0.06 (0.05–0.07)	0.06 (0.04–0.12)	0.14 (0.10–0.40)	0.24 (0.16–0.73)
Extrathoracic region	0.00 (0–0.01)	0 (0–0.01)	0.01 (0.01–0.03)	0.02 (0.02–0.05)
Muscle	0.00 (0–0.01)	0.01 (0–0.01)	0.01 (0.01–0.02)	0.02 (0.02–0.08)
Oral mucosa	0.01 (0.01–0.01)	0.01 (0.01–0.02)	0.02 (0.02–0.02)	0.04 (0.02–0.12)

RBM: red bone marrow.

### KAP and FT

No significant differences were found in KAP and FT values among the groups based on ΒΜΙ, gender, type of fusion and type of implants used (*p* > 0.05) (Table 3). The latter finding demonstrates the increased neurosurgeons’ familiarisation with surgical technique and the selection of the technical parameters of the C-arm, keeping the radiation exposure as low as reasonably practicable^(23)^. Regarding the treated levels, a significant difference was found in KAP values between C4/C5 and C5/C6 levels (*p* = 0.032), although FT differences between these groups were not significant (*p* = 0.72).

A recent four-centre study in France found a significant variability in KAP and FT values, although two experienced surgeons performed the ACDFs in each centre. The KAP and FT were 0.578 Gycm^2^ and 24.0 s, 0.059 Gycm^2^ and 11.0 s, 0.175 Gycm^2^, and 0.196 Gycm^2^ and 28.8 s for centres 1, 2, 3 and 4, respectively. This was concluded to depend on the fluoroscopy equipment, surgical practice and exposure parameters. The mean KAP and FT values were 0.357 Gycm^2^ and 19.7 s for all centres^(24)^. The mean KAP (0.15 Gycm^2^) and FT (6.7 s) values in the current study are 2.4- and 3-fold lower than these values. Fransen^(8)^, Crawley *et al*.^(7)^, Lee *et al*.^(11)^ and Metaxas *et al*.^(12)^ reported FT and KAP values for ACDF procedures. The mean FT values in these studies were 0.17, 0.80 (median), 0.16 and 0.12 min, respectively. The corresponding mean KAP values were 0.17, 0.42 (median), 0.20 and 0.21 Gycm^2^, respectively. In another study, McCormick *et al*.^(25)^ concluded that BMI does not appear to impact the FT during cervical interlaminar injections significantly. They reported that the FT is significantly longer only for obese compared with overweight patients and not for obese compared with normal patients. However, in this study, there is no significant difference in dose data among the BMI groups investigated.

### Cumulative and incident air-kerma (*K*_air_)

No significant differences were found in incident *K*_air_ values regarding the groups based on BMI, gender, type of fusion and type of implants used (*p* > 0.05) (Table 3). Regarding the treated levels, a significant increase in incident *K*_air_ was only found in C5/C6 compared with C4/C5 levels (*p* = 0.030). This finding is probably attributed to the significantly higher KAP required in C5/C6 than in C4/C5 levels (*p* = 0.032).

During an ACDF procedure, the IRP does not coincide with the patient’s entrance, and the displayed *K*_air_ may lead to an underestimation of the actual air-kerma incident on the patient. Using the inverse square law between the IRP, which is located 69.5 cm from the X-ray focus, and the FSD, a total mean incident *K*_air_ of 1.04 mGy (range, 0.02–10.10) was found.

Previous studies have reported dosimetric data during cervical spine interventions^(7–12, 14, 15)^; however, there are only a few data regarding their association with patient- and procedure-related data^(13, 14)^. Giordano *et al*. reported a mean patient entrance dose of 164 and 98 mGy during a 5-min fluoroscopic exposure of a cadaveric cervical spine specimen utilising a standard^(9)^ and a mini-C-arm^(10)^ with a tube voltage of 78 and 75 kVp, respectively. Tsalafoutas *et al*.^(15)^ reported a mean patient entrance dose of 173 mGy during a 4.2-min bilateral cervical pedicle screw placement using a mean tube voltage of 76 kVp. However, the FT in these studies is much higher, showing that ACDFs could result in significantly greater radiation doses than those reported in this study, if the neurosurgeons use prolonged fluoroscopic exposures. Metaxas *et al*.^(12)^ also reported a mean patient entrance dose of 1.95 mGy during ACDF procedures with a mean tube voltage of 65 kVp.

### Peak skin dose (PSD)

**Figure 2:**
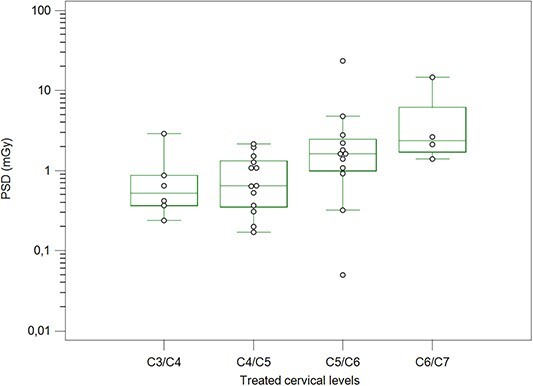
Box plots of PSDs for the different single cervical levels treated during ACDF procedures

**Figure 3:**
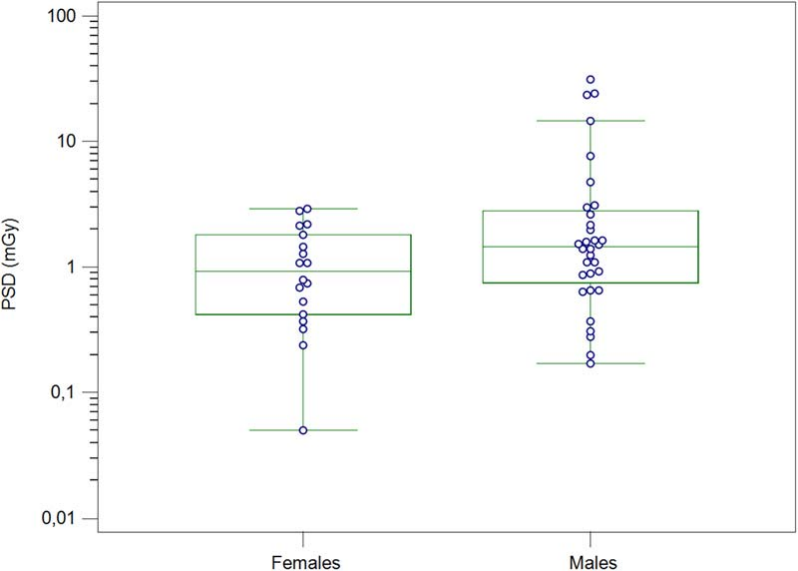
Box plots of PSDs for the male and females undergoing ACDF procedures

The PSD is also reported by the VirtualDose-IR software^(20)^. The mean PSD for the total group of patients was 3.20 mGy (range, 0.05–31.20). The incident *K*_air_ and PSD values are not similar. Still, the PSD is higher about a factor of 3 mainly due to the calculation method of PSD by the VirtualDose-IR software and that incident *K*_air_ is the air kerma measured in the air without including backscattered radiation from the patient as the PSD. A significant difference was found among the cervical levels (*p* = 0.036). Figure 2 shows the comparisons of PSDs among these groups. Pairwise comparisons showed only a significant difference in PSD between C6/C7 and C4/C5 levels (*p* = 0.013). The males received 274% higher mean PSD than females (Figure 3). However, the difference was insignificant (*p* = 0.12) due to the outliers observed in male patients resulting in relatively higher mean values for this group than the median values (0.94 mGy for females and 1.46 mGy for males).

Using the KAP conversion factors (males: 4.369 mGyGycm^−2^, females 4.354 mGyGycm^−2^) reported by Metaxas *et al*.^(12)^, the maximum skin dose for males was 6% lower than for females (0.66 and 0.70 mGy for males and females, respectively). This is probably due to the differences between the software and the phantoms used in the simulations. In summary, the maximum skin doses during ACDF procedures are well below the threshold entrance skin dose of 2 Gy for deterministic effects (skin erythema)^(2, 5)^ regardless of the three dose metrics used: cumulative *K*_air_, incident *K*_air_ and PSD calculated by VirtualDose-IR.

### Effective dose (E)

No significant differences were found in *E* values regarding the groups based on BMI, gender and type of implants used (*p* > 0.05) (Table 3). A significant difference was found between single- and multi-level fusions (*p* = 0.037). Notably, a significant difference was found in *E*, although both KAP and FT values are not significantly different between these groups. The *E* values also showed significant differences among the cervical levels (*p* = 0.007). Pairwise comparisons of *E* having the least *p*-values included those between the C6/C7 and C4/C5 groups (*p* = 0.007) and between the C5/C6 and C4/C5 groups (*p* = 0.012). Notably, a significant difference was found in *E* between C6/C7 and C4/C5 groups, although both KAP and FT values are not significantly different between these groups.

ACDF procedures result in a slight variation in the patient’s dose due to very different imaging conditions than those associated with thicker regions, such as the lumbar spine^(13, 16, 17)^. Notably, the *E* is extremely lower compared with other spine interventional procedures, such as those performed in the lumbar spine^(11, 13, 16)^. Indicatively, the *E* in ACDF is similar to that resulting from a lateral X-ray radiograph of the cervical spine^(26)^. However, even if the *E* is very low, it should not be ignored, no matter how small, because any dose could have adverse health effects^(2)^.

Crawley *et al*.^(7)^ and Metaxas *et al*.^(12)^ calculated an *E* of 0.07 mSv using the ODS60 software and 0.015 mSv using the CALDoseX software^(27)^, respectively. Lee *et al*.^(11)^ calculated an *E* of 0.04 mSv using a KAP-to-*E* conversion factor of 0.2 mSvGy^−1^cm^−2^. The mean *E* in this study (0.023 mSv) is 67 and 43% lower than Crawley *et al*.’s^(7)^ study and Lee *et al*.’s^(11)^ study and 53% higher than Metaxas *et al*.’s^(12)^ respective values.

Chin *et al*.^(28)^ reported mean radiation doses for single- or multi-level fusions in two groups: fusions with cages (group 1) and fusions with plates and screws (group 2). The mean *E* was 0.8 and 1.3 mSv in groups 1 and 2, respectively. A significant difference was reported between these groups, but this difference did not reach statistical significance in this study. For single-level fusions, a mean *E* of 0.5 and 1.2 mSv was reported for groups 1 and 2. For two-level fusions, the mean *E* was 0.9 and 1.4 mSv for groups 1 and 2, respectively^(28)^. A significant difference in *E* was reported for single- and two-level fusions between the two groups, while in our study, the corresponding differences between the same groups for single- and multiple-level fusions were not statistically significant (*p* = 0.37; *p* = 0.30). Notably, patients’ age and BMI (Table 2) were almost similar for the two studies; however, no significant difference was demonstrated between the two groups in the two studies.

In a previous study, using computed tomography navigation increased the radiation exposure to the patient and reduced the radiation exposure to the surgeon. In addition, thoracic and lumbar interventions had higher *E* than cervical interventions (6.93 vs 2.34 mSv)^(29)^. However, in this study, the *E* due to fluoroscopy for ACDF was 102-fold lower than CT-based navigation procedures in the cervical region. Regarding single cervical interlaminar injections, using pulsed or low-dose fluoroscopy instead of standard continuous fluoroscopy resulted in a 30% reduction of FT^(30)^.

### Organ doses (ODs)

Tables 4–6 present an overview of the median ODs for the total patient sample that underwent ACDF procedures as a function of patients’ BMI, gender, type of fusion, cervical levels and type of implants used. The urinary bladder, breasts, colon, gonads, liver, stomach, adrenals, gallbladder, kidneys, eye lenses, pancreas, small intestine, spleen, brain, heart, thymus, lymphatic nodes and prostate/uterus did not absorb any OD (0 mGy). These organs are located far away from the primary X-ray beam (Figure 1). The organs that received the largest doses in all groups studied were the thyroid, oesophagus and salivary glands (Tables 4–6) due to that these organs are very close to the primary X-ray beam (Figure 1). Neurosurgeons should always consider the dose absorbed by the thyroid, an organ with high radiosensitivity. When younger patients with many years of life expectancy are involved, this is especially important since this organ receives the largest dose during ACDF (Tables 4–6). An increase of 33% was found only in salivary glands’ doses for overweight than normal patients. On the other hand, a decreasing trend of up to 100 and 50% was observed for doses received by the organs of overweight and obese patients compared with normal patients (Table 4). Additionally, an increase in median ODs was found for male than female patients (except the lungs) (Table 4) and when the fusions were performed to C5/C6 or C6/C7 compared with C3/C4 or C4/C5 levels (Table 6). For example, the median thyroid dose of an overweight and an obese patient was 60 and 25% lower than for a normal patient, respectively. Additionally, the median thyroid dose for a male patient was 15% higher than for a female patient. Furthermore, a C6/C7 procedure resulted in a 277 and 308% higher thyroid dose than C3/C4 and C4/C5 levels, respectively, while a procedure in C5/C6 resulted in a 108 and 125% higher thyroid dose than C3/C4 and C4/C5 levels. However, no significant differences were found in all ODs among BMI groups (*p* > 0.05) (Table 4). Only the salivary glands’ dose showed a significant difference between male and female patients (*p* < 0.001) (Table 4), which is due to the largest part of this organ that is inside the X-ray field during irradiations of the males’ phantoms. Regarding the cervical levels, a significant difference was found for the bone surface (*p* = 0.018), red bone marrow (RBM) (*p* = 0.013), oesophagus (*p* = 0.020), thyroid (*p* = 0.024), extrathoracic region (*p* = 0.009) and oral mucosa (*p* = 0.027) (Table 6). The pairwise comparisons between C3/C4 and C4/C5 with C5/C6 and C6/C7 gave rise to the difference in bone surface’ and RBM doses (*p* < 0.05). The difference in oesophagus, thyroid and extrathoracic region doses was due to the differences in pairwise comparisons between C3/C4 and C4/C5 with C6/C7 levels, as well as C4/C5 and C5/C6 levels, while for oral mucosa dose in the differences only between C3/C4 and C4/C5 with C6/C7 levels (*p* < 0.05).

An increasing trend of up to 100% (oesophagus, extrathoracic region) was also found in median ODs for multiple-level compared with single-level fusions. However, a significant increase was observed only for the extrathoracic region (*p* = 0.029) (Table 5). Regarding the type of implants, fusion with cages plus plates and screws resulted in higher ODs (up to 100%) (oesophagus) than the fusions with a cage as a stand-alone device. However, a significant increase was found only in ODs for muscle (*p* = 0.041) (Table 5).

In a previous study, Metaxas *et al*.^(12)^, using CALDoseX software^(27)^, reported OD conversion coefficients normalised over KAP values for patients undergoing an ACDF procedure. ODs could also be calculated by multiplying these conversion coefficients and the mean total KAP of 0.15 Gycm^2^. There are substantial increases for the RBM, bone surface and skin doses calculated with conversion coefficients provided by the CALDoseX software than VirtualDose-IR. These differences are due to different approaches used for calculating these ODs for organs distributed throughout the human body and are only partially irradiated during ACDF. The CALDoseX software^(27)^ calculates the doses for these organs in a 7.2 × 7.2 cm^2^ area along the central axis of the X-ray beam, where it enters the phantom. In both studies, the thyroid receives a relatively large dose regarding its radiosensitivity. The mean thyroid dose in this study (0.290 mGy) is 110% higher than that obtained from the previous study (0.157 mGy)^(12)^. Additionally, 21–72% lower ODs were calculated in this study regarding the brain, oral mucosa, salivary glands, extrathoracic region and lymphatic nodes, while 25–70% higher ODs were found for the thymus, lungs and heart. Furthermore, in this study, a substantially higher dose (992%) was calculated for the oesophagus due to differences in the design of the two phantoms considering the variations in the organs’ position, shape and size and exposure geometry used in the simulations.

### Optimisation

The key points to manage radiation dose during ACDF procedures include the following: know the settings of the C-arm, maximise the FSD, minimise the FT, keep a record of FT, KAP and *K*_air_ for each patient, use pulsed fluoroscopy with the lowest frame rate that provides consistent image quality, be aware that lateral projections result in a higher dose, vary the position where the beam enters the patient to avoid maximising the PSD, minimise the number of CINE runs or frames, avoid using electronic magnification, collimate the X-ray field, use of ABC and the saved images instead of acquiring additional radiographs^(6, 16)^. Neurosurgeons should also receive training in radiation protection issues^(2, 3)^. The reported radiation doses in this study are dependent of the standard routine fluoroscopy method used. At the end of the study, a medical radiation physicist in our Department presented the study’s results in a workshop and informed neurosurgeons regarding the collimation and other practical options for manipulation of the C-arm to reduce radiation dose in the future.

### Limitations

One of the study’s main limitations is using one C-arm equipped with an image intensifier in a single hospital. Additionally, round tube voltage values and a square X-ray field, rather than the circular one employed in clinical practice, were used as input in the simulations. However, the difference in the shape of the X-ray field between clinical practice and the simulations may result in an underestimation or overestimation of ODs for the organs located at the boundaries of the X-ray field. Additionally, the fixed position of the X-ray field during the simulations may result in differences among the organs clinically exposed and those irradiated during the simulations, adding additional uncertainties (although very small) in OD calculations. The selection of a fixed FSD value of 56 cm may result in an underestimation or overestimation of incident *K*_air_ and PSD values. For an FSD of 54 cm, the mean incident *K*_air_ and PSD values are overestimated ~8% (mean 1.12 mGy, range 0.02–10.90) and 20% (mean 3.84 mGy, range 0.06–37.4), while for an FSD of 58 cm are underestimated ~7% (mean 0.97 mGy, range 0.01–9.45) and 18% (mean 2.63 mGy, range 0.04–25.7) compared with those calculated with an FSD of 56 cm and the same exposure and operative parameters. Nevertheless, considering the ODs, PSD and *E* received by the patients during ACDF procedures, the estimated dose values could still be considered conservative despite all the above limitations. However, further research is necessary to acquire more representative dose values, including patients from several hospitals, flat-panel detector C-arms and neurosurgeons with varying experience levels.

## Conclusion

This study reported ODs, PSD and *E* during ACDF procedures based on patient-related (BMI, gender) and procedure-related data (single- or multi-level fusion, fusion using cages or cages plus plates and screws, fusion in C3/C4 or C4/C5 or C5/C6 or C6/C7 levels) utilising VirtualDose-IR software. The BMI, gender and type of implants did not significantly impact KAP, incident *K*_air_, PSD and *E* values. However, the type of fusion significantly affected the *E*. The fusions performed in C5/C6 resulted in significantly higher KAP, incident *K*_air_ and *E* values compared with C4/C5 levels, while the *E* and PSD for C6/C7 are significantly higher than those for C4/C5 levels. The organs that received the highest doses in all groups studied were the thyroid, oesophagus and salivary glands. The BMI did not have a significant impact on the ODs. However, the salivary glands’ dose significantly increased in male patients, and the extrathoracic region’s dose significantly increased for multi-level fusions. In contrast, C6/C7 fusions resulted in significantly higher oesophagus and thyroid doses than C3/C4 and C4/C5 levels and fusions in C5/C6 compared with C4/C5 levels. The dosimetric data presented here could be helpful for neurosurgeons as a comparator with other Hospitals’ practices or with future studies in our Hospital in optimising exposure technique during ACDF procedures by keeping the ODs, PSD and *E* as low as reasonably achievable. However, additional studies need to be conducted to evaluate further the effect of other potential clinical or technical factors on ODs.

## Data Availability

The data that support the findings of this study are available from the corresponding author upon reasonable request.
